# Shoulder-specific rehabilitation combined with aerobic exercises versus solely shoulder-specific rehabilitation in patients with type 2 diabetes mellitus: study protocol for a randomized controlled superiority trial

**DOI:** 10.1186/s13063-022-06647-5

**Published:** 2022-08-17

**Authors:** Fernanda A. P. Habechian, Mauricio E. Flores Quezada, Ann M. Cools, Birgitte Hougs Kjaer, Rodrigo I. Cuevas Cid, Gisele G. Zanca

**Affiliations:** 1 Laboratory of Clinical Research in Kinesiology, Department of Kinesiology, Universidad Católica del Maule, Casa Central: Avda. San Miguel, 3605 Talca, Chile; 2 Faculty Medicine and Health Sciences, Department of Rehabilitation Science and Physiotherapy, Ghent University, Campus Heymans (UZ Ghent), Building B3 - Second floor, De Pintelaan 185, 9000 Ghent, Belgium; 3 Department of Physical and Occupational Therapy, Bispebjerg and Frederiksberg University Hospitals, Bispebjerg Bakke 23, DK-2400 Copenhagen, NV Denmark; 4 Postgraduate Program in Aging Sciences and Postgraduate Program in Physical Education, São Judas Tadeu University, Rua Taquari, 546. Mooca, São Paulo, 03166-000 Brazil

**Keywords:** Shoulder pain, Diabetes mellitus type 2, Musculoskeletal rehabilitation, Randomized controlled trial

## Abstract

**Background:**

Musculoskeletal disorders are very common in patients with diabetes mellitus (DM). The upper limb is one of the regions that is most frequently affected generally presenting limited joint mobility, pain, and a decreased muscle strength. Most clinical trials with a focus on shoulder musculoskeletal rehabilitation are carried out in patients who do not present DM. Thus, the purpose of the present study is to compare the effects of two distinct treatment protocols (conventional shoulder musculoskeletal rehabilitation combined with aerobic exercises versus solely conventional shoulder musculoskeletal rehabilitation) on shoulder pain, function, strength, kinematics, and supraspinatus tendon thickness in patients with type 2 DM after 12 weeks of intervention and a subsequent follow-up at week 20.

**Methods:**

A randomized controlled superiority trial will be conducted. Participants with a clinical diagnosis of type 2 DM of both sexes, age between 40 and 70 years, presenting shoulder pain will be randomly assigned to one of the following groups: (1) conventional shoulder musculoskeletal rehabilitation combined with aerobic exercises; (2) solely conventional shoulder musculoskeletal rehabilitation. All individuals will be evaluated before starting the treatment protocol (baseline) and at the end of treatment (post 12 weeks) and as a follow-up at 20 weeks. The shoulder function assessed by the SPADI (Shoulder Pain and Disability Index) questionnaire will be considered as primary outcome; the secondary outcome will be shoulder pain, measured with NPRS scales. Other outcomes will include range of motion, measured using a digital inclinometer; isometric shoulder muscle strength, measured using a manual muscle dynamometer; shoulder kinematics, measured using three-dimensional inertial units measurement; supraspinatus tendon thickness, measured using an ultrasound; AGE accumulation, using a skin autofluorescence measurement; and HbA1c (hemoglobin a1c), fasting glucose and lipid profile measured by a simple blood test.

**Discussion:**

DM is a highly prevalent disease and a public health problem worldwide, and the upper extremity musculoskeletal disorders in DM are barely recognized and largely underestimated. In this way, it would be interesting to analyze if the combination of aerobic exercises with conventional musculoskeletal rehabilitation protocols could generate better results in the functionality, pain, mobility and an improvement in the biochemical aspects related to the hyperglycemia of these patients compared to solely the conventional musculoskeletal rehabilitation.

**Trial registration:**

ClinicalTrials.gov NCT04817514. Registered on March 26, 2021.

## Administrative information

**Table:** 

Title {1}	Shoulder-specific rehabilitation combined with aerobic exercises versus solely shoulder-specific rehabilitation in patients with type 2 Diabetes Mellitus: study protocol for a randomized controlled superiority trial
Trial registration {2a and 2b}.	ClinicalTrials.gov Identifier: NCT04817514
Protocol version {3}	Version 1 (November 22, 2021) Version 2 (June 25, 2022)
Funding {4}	National Fund for Scientific and Technological Development of Chile (Fondecyt - ANID, Grant #11200574) – financial support
Author details {5a}	**Fernanda A.P. Habechian:** fernanda.aph@gmail.com. Departamento de Kinesiología, Facultad de Ciencias de la Salud, Universidad Católica del Maule, Casa Central: Avda. San Miguel 3605, Talca, Chile. **Mauricio E. F. Quezada:** mefquezada@gmail.com. Departamento de Kinesiología, Facultad de Ciencias de la Salud, Universidad Católica del Maule, Casa Central: Avda. San Miguel 3605, Talca, Chile. **Gisele Garcia Zanca :** gisele_gz@yahoo.com.br. Postgraduate Program in Aging Sciences and Postgraduate Program in Physical Education. São Judas Tadeu University. Rua Taquari, 546. Mooca, São Paulo, São Paulo. Zip Code: 03166-000 **Ann M. Cools:** ann.Cools@ugent.be. Faculty Medicine and Health Sciences Department Rehabilitation Sciences and Physiotherapy. Campus Heymans (UZ Ghent), Building B3 - Second floor De Pintelaan 185, 9000 Gent Belgium. **Birgitte Hougs Kjaer:** birgitte.hougs.kjaer@regionh.dk. Department of Physical and Occupational Therapy Bispebjerg and Frederiksberg Hospitals. Bispebjerg Bakke 23, DK-2400 Copenhagen NV, Denmark. **Rodrigo I. Cuevas Cid:** rodrigo.cuevas@alu.ucm.cl. Departamento de Kinesiología, Facultad de Ciencias de la Salud, Universidad Católica del Maule, Casa Central: Avda. San Miguel 3605, Talca, Chile. FAPH is the grant holder and conceived the study and with MEFQ, RICC initiated and implemented the study. GGZ, AMC and BHK provided statistical and methodological expertise in clinical trial design. All authors contributed to refinement of the study protocol and approved the final manuscript.
Name and contact information for the trial sponsor {5b}	Trial Sponsor investigator: Fernanda A. P. Habechian Address: Avda. San Miguel 3605, Talca, Chile. Universidad Católica del Maule Email: Fernanda.aph@gmail.com
Role of sponsor {5c}	Main researcher, conceived, initiated and implemented the study.

## Background and rationale {6a)

Diabetes mellitus (DM) has become a public health problem, since approximately 537 million adults worldwide are living with the disease in 2021, and it is estimated that this number will increase to approximately 784 million adults worldwide in 2045 [1]. Chile ranks as the second country in South America with the highest prevalence of DM, with an estimation of 1,262,200 people, which represents 9.8% of the adult population aged 20–79 years [1].

Besides the most reported complications of DM, such as cardiovascular disorders and peripheral neuropathy, musculoskeletal disorders are also a very common finding in this population [2, 3]. These disorders receive relatively little attention [4, 5]; however, they result in physical and psychological harm in people with DM, compromising their quality of life. Upper limbs are frequently affected in this population, generally presenting limited joint mobility, pain, and decreased muscle strength [2–6]. According to the literature, individuals with DM present 27.5% of prevalence of shoulder complex disorders, such as rotator cuff injuries and adhesive capsulitis, compared to only 5% in the non-diabetic population [5, 6].

The mechanisms of upper extremity musculoskeletal injuries in subjects with DM are still not clear. Some evidence suggests that accumulation of the non-enzymatic advanced glycation end-products (AGEs), due to hyperglycemia, increases the crosslinking in collagen [7]. This increase can generate abnormal collagen deposits in tendons, ligaments, and skin, making connective tissues thicker, rigid, and weaker, leading to a diffuse arthrofibrosis [2, 8–10].

The literature shows that patients with DM and adhesive capsulitis present a greater difficulty in achieving the desired results in their musculoskeletal rehabilitation protocol compared to patients without DM [11–13]. This could be explained by the fact that the mechanisms related to musculoskeletal injuries in patients with DM are different from the general population and that the rehabilitation protocols are usually focused on the symptomatic structure without considering the systemic disease [12, 13]. A wide range of studies have demonstrated that aerobic exercises are the most effective way for glycemic control, reducing cardiovascular risk factors, contributing to weight loss and well-being [14, 15]. Thus, aerobic exercise could be an important complementary strategy in musculoskeletal rehabilitation protocols for patients with DM. In addition, most clinical trials with a focus on shoulder musculoskeletal rehabilitation are carried out on patients who do not present DM or only patients with a specific disorder such as adhesive capsulitis, not considering other shoulder dysfunctions. Research in this area is scarce, perhaps because other diabetes complications have been considered more life-threatening and more important, such as cardiovascular disease. However, musculoskeletal disorders may decrease physical mobility and represent a substantial burden on morbidity and on the quality of life of this population.

Therefore, research is needed to better understand whether the addition of aerobic exercises to a standard shoulder rehabilitation program could help reduce musculoskeletal complications in this population.

## Objectives {7}

The main purpose of this study is to compare the effects of two distinct treatment protocols (conventional shoulder musculoskeletal rehabilitation combined with aerobic exercises versus solely conventional shoulder musculoskeletal rehabilitation) on shoulder function, range of motion (ROM), pain, strength, kinematics, and supraspinatus tendon thickness in patients with type 2 DM after 12 weeks of intervention, and a subsequent followup at the week 20 (8 weeks after the end of the protocol). As a secondary objective, we will evaluate the association between AGE accumulation and shoulder pain, function, strength, kinematics, and supraspinatus tendon thickness in individuals with type 2 DM.

The hypothesis is that the addition of aerobic exercises to the shoulder-specific rehabilitation protocol will promote a greater decrease in shoulder pain and increase in functionality, range of motion, strength, and an adequate shoulder kinematics after 12 weeks, and a follow-up at week 20. Furthermore, regarding the association, the hypothesis is that a higher AGE accumulation is associated with higher shoulder pain, dysfunction, tendon thickness, shoulder kinematic disorders, and lower muscle strength in patients with type 2 DM.

## Trial design {8}

This is a randomized controlled superiority trial, single-blind, two-arm and parallel-group study. Participants will be randomly assigned to one of the following groups: (1) conventional shoulder musculoskeletal rehabilitation combined with aerobic exercises; (2) solely conventional shoulder musculoskeletal rehabilitation.

## Methods: participants, interventions, and outcomes

### Study setting {9}

The study will be performed in the Laboratory of Clinical Research in Kinesiology from the Universidad Católica del Maule – Talca, Chile.

### Eligibility criteria {10}

#### Inclusion criteria

The inclusion criteria will be participants with a clinical diagnosis of type 2 DM (with at least 1 year of diagnosis), of both sexes, between 40 and 70 years old, presenting shoulder pain (unilateral or bilateral) for at least 3 months with a pain intensity score from 3 points on a numerical pain rating scale (NPRS).

#### Exclusion criteria

Participants will be excluded if they present a history of shoulder surgery; recent history of fracture in the upper limb; cognitive deficits that make it difficult to understand verbal commands; neuromuscular diseases; central nervous system diseases; diagnosis of cardiovascular disease; kidney chronic disease and have undergone shoulder rehabilitation within the past 6 months

### Who will take informed consent? {26a}

The MFQ and RIC researchers will be in charge of contacting the participants, inviting them to participate in the study. The researchers will explain the objectives of the study and ethical implications, followed by evaluation according to the eligibility criteria. If the participant is eligible and accepts to participate in the study, the informed consent will be signed in 2 copies: one for the participants, and one for the researcher.

### Additional consent provisions for collection and use of participant data and biological specimens {26b}

This is not applicable, since this study does not involve any biological specimens.

## Interventions

### Explanation for the choice of comparators {6b}

Considering that the majority of the protocols for shoulder rehabilitation only focus on the musculoskeletal disorder, without considering the presence of metabolic diseases, the rehabilitation protocols to be compared are the following: (1) a protocol only focused on shoulder rehabilitation (shoulder-specific rehabilitation protocol); (2) a protocol of shoulder rehabilitation associated to an aerobic exercise, with an emphasis in the metabolic disease (shoulder-specific rehabilitation combined with aerobic exercises protocol).

### Intervention description {11a}

Participants who meet the inclusion criteria for this study will initially be evaluated for demographic data including age, sex, weight, height, occupation, and time since type 2 DM diagnosis. The order of assessment of the main outcomes will be randomized. After participants complete the outcomes assessment, they will be randomly assigned to one of the two treatment groups: (1) shoulder-specific rehabilitation protocol group (SRG); or (2) shoulder-specific rehabilitation protocol plus aerobic exercise group (SAG). The randomization sequence will be performed through a computerized algorithm in GraphPad.

All measurements will be performed on the symptomatic side for shoulder pain. In the case of bilateral pain, the side with greater severity will be considered in the study as proposed in previous studies [16]. However, treatment will be carried out bilaterally. Outcomes measures will be performed always by the same evaluator.

**Fig. 1:**
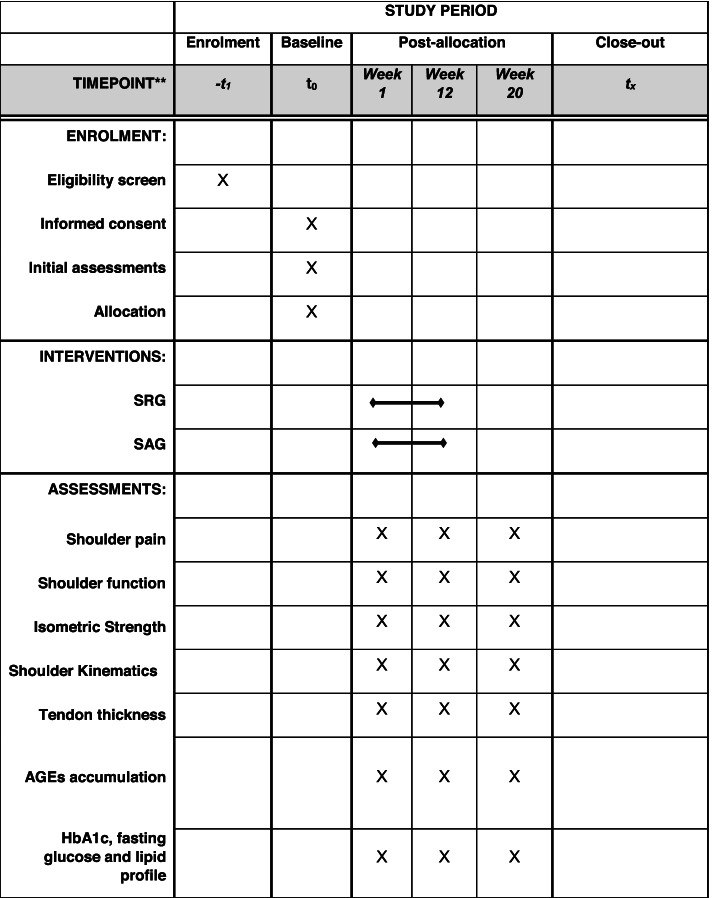
The recommended SPIRIT figure with the participant timeline

Both treatment protocols will last 12 weeks, and they will be performed twice a week. All individuals will be evaluated before starting the treatment protocol (baseline), at the end of treatment (post 12 weeks), and at week 20 (8-week follow-up after the intervention) (Fig. 1). To minimize bias, the evaluator responsible to measure the main outcomes will be blinded to the type of treatment being performed. Before, during, and after each session, blood pressure, heart rate, and rating of perceived exertion will be measured.

#### SRG: shoulder-specific rehabilitation group

The SRG protocol will be performed on groups with a maximum of 5 participants. Exercise intensity and difficulty will progress after completing 4 weeks of treatment, by increasing range of motion and loads (dumbbells) or resistance (elastic bands) in the exercises that are convenient. Each session will consist of 6 exercises: (1) pendulum exercise (10 repetitions—maintenance for 20 s); (2) slide in flexion of the arms on the table (2 sets of 10 repetitions, maintenance in maximum range for 20 s); (3) abduction and adduction mobility (2 sets of 10 repetitions, maintenance for 20 s) and full can abduction exercise; (4) wall slide—sliding of the arms on the wall (2 series of 10 repetitions, maintenance of 20 s); (5) internal and external rotation mobility (2 sets of 10 repetitions, maintenance for 20 s) and external rotators strengthening; (6) stretching of the posterior capsule (cross-body) and stretching of pectoralis minor (3 repetitions of 30 s, each). Pain will be evaluated during the protocol using the NPRS scale. The exercises proposed in this protocol are those already demonstrated to improve function, strength, range of motion, and pain in musculoskeletal disorders of the shoulder complex for general population [17, 18].

Each session of this protocol will last between 30 and 35 min. All sessions will be supervised by a physiotherapist with experience in physical exercise.

#### SAG: shoulder-specific rehabilitation combined with aerobic exercises group

The SAG will perform the shoulder rehabilitation protocol presented above combined with an aerobic exercise program. The aerobic program will last 20 min per session at 40% of the reserve heart rate (HRR), progressing up to 40 min with a maximum of 60% of the HRR in the last 2 weeks (weeks 11 and 12), according to the recommendations for patients with type 2 DM proposed by the American College of Sports Medicine [19]. The program will be carried out in groups of 5 participants and each session will consist of 3 stages: (1) warm-up (5 min): patients will perform stretches of the main muscle groups; (2) aerobic exercise on a treadmill with continuous heart rate monitors (Polar Electro Oy, Kempele, Finland), which will be used to adjust workload to achieve the target heart rate (15–40 min); (3) cooldown (5 min): stretching of the main muscle groups which were worked during the sessions and relaxation. In this way, the rehabilitation protocol of SAR group will last a total of approximately 90 min maximum. During sessions, heart rate will be monitored by a heart rate monitor (Polar Vantage, Finland), and rating of perceived exertion will be measured using the Borg CR20 scale, which should remain approximately between 11 and 13 [19]. All sessions will be supervised by a physiotherapist with experience in physical exercise.

### Criteria for discontinuing or modifying allocated interventions {11b}

Participants will be informed that exercises and rehabilitation interventions, other than those that will be received during the duration of the study, will not be allowed. Participants with less than 80% treatment attendance will be excluded from the study. Furthermore, data such as blood pressure, heart rate, saturation, and rating of perceived exertion will be measured before, after, and during the rehabilitation sessions. Capillary blood glucose will be evaluated at the beginning and at the end of each session. If the patient presents signs or symptoms of decompensation such as glycemia> 200 mg/dl or <80 mg/dl, systolic blood pressure > 180 mmHg, or diastolic blood pressure > 100 mmHg at rest, the session or evaluation will be immediately suspended, and the necessary measures will be taken.

### Strategies to improve adherence to interventions {11c}

The researchers will ask for feedback from the patients throughout the sessions, to know if there is anything that could be improved, and about the patient’s satisfaction. If they notice anything that could help to improve their adherence, it will be added, without changing the intervention protocol. Furthermore, the patients will be reminded weekly about their rehabilitation sessions through messaging.

### Relevant concomitant care permitted or prohibited during the trial {11d}

Participants will be instructed to keep their pharmacological treatment during the entire study and inform researchers of any changes in their medications. Participants will be informed that exercises and rehabilitation interventions, other than those that will be received during the duration of the study, will not be allowed.

### Provisions for post-trial care {30}

At the end of the study, if SAG protocol is effective in promoting higher improvement than the SRG protocol, the same treatment will be offered to SRG participants at no cost**.**

### Outcomes {12}

The results will be evaluated at 3 points: (1) baseline (before starting the intervention program), (2) after 12 weeks of intervention (after 48 h to 1 week from the last intervention), and (3) at the week 20 (follow-up). Regarding the outcomes, the shoulder assessed by the SPADI (Shoulder Pain and Disability Index) questionnaire will be considered as primary outcome; the secondary outcome will be shoulder pain, measured with NPRS scales. Other outcomes will include range of motion, measured using a digital inclinometer; isometric shoulder muscle strength, measured using a manual muscle dynamometer; shoulder kinematics, measured using three-dimensional inertial units measurement; supraspinatus tendon thickness, measured using an ultrasound; AGE accumulation, using a skin autofluorescence measurement; and HbA1c (hemoglobin a1c), fasting glucose and lipid profile measured by a simple blood test.

### Participant timeline {13}

#### Sample size {14}

Sample size calculation was performed through the GPower software (version 3.1). The calculation was based on the study by Schmitt et al. [20], using the main outcome (SPADI questionnaire) to detect a mean difference of 16.8 points, considering a power of 80% and an Alfa of 5%. The sample size suggested was 16 per group; however, to account for possible withdrawals that may occur in this type of study, it will be increased by 20%, thus totaling 40 participants (20 per group).

#### Recruitment {15}

Participants will be recruited from the local community through posters, leaflets distributed on university premises, health service centers in the city of Talca, and physiotherapy clinics in the region. Recruitment will also occur through social media and personal invitation.

## Assignment of interventions: allocation

### Sequence generation {16a}

A researcher will generate the sequence number through a randomization on the GraphPad software. Participants will be randomly assigned, in a 1:1 ratio, to one of the two groups studied (1) SRG or (2) SAG.

### Concealment mechanism {16b}

After the sequence is generated, sealed envelopes containing the numbers “1” or “2” will be prepared according to the predefined sequence.

### Implementation {16c}

**Fig. 2:**
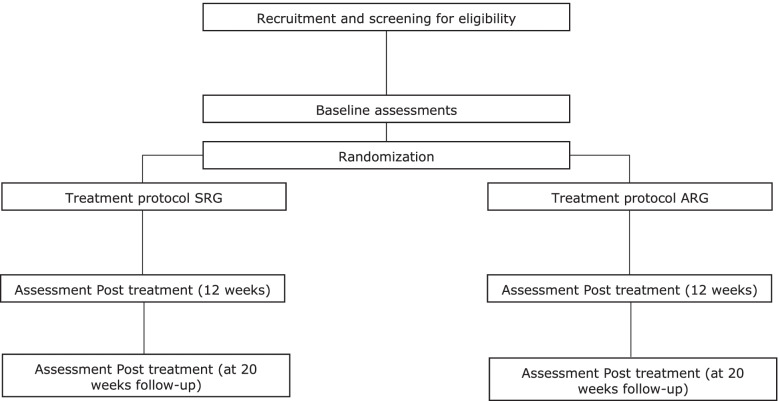
Flowchart illustrating the experimental design of the present study

The allocation sequence will be generated by a researcher blinded to the study protocol. Sealed and opaque envelopes in numerical order will be ready to be used after the baseline assessments, when the envelop will be unsealed by the researcher MFQ. Figure 2 presents a flowchart illustrating the experimental design of the present study.

## Assignment of interventions: blinding

### Who will be blinded {17a}

This will be a single-blinded study. The researchers responsible for outcome assessments, data entry, and statistical analysis will be blinded to the group assignments; they will not have access to the patient’s rehabilitation place, nor to any information related to the intervention.

### Procedure for unblinding if needed {17b}

There would be no reason for unblinding, since only the researcher responsible for outcome assessment and data analysis will be blinded.

## Data collection and management

### Plans for assessment and collection of outcomes {18a}

For shoulder pain all measurements will be performed on the symptomatic side. In the case of bilateral pain, the side with greater severity will be considered in the study as proposed in previous studies [16]. However, treatment will be carried out bilaterally. To minimize bias, the evaluator responsible to measure the outcomes will be blinded to the type of treatment performed. The assessments and treatment protocols will be carried out in the Clinical Research Laboratory in Kinesiology at the university. Data such as blood pressure, heart rate, saturation, and rating of perceived exertion will be measured before, after, and throughout the rehabilitation sessions. If the patient presents signs or symptoms of hypoglycemia; glycemia > 200 mg/dl or <80 mg/dl; systolic blood pressure > 180 mmHg, or diastolic blood pressure > 100 mmHg at rest, the session or evaluation will be immediately suspended, and the necessary measures will be taken. The same evaluator will perform all the tests and evaluative measures of the variables described below.

#### Shoulder pain measurement

Shoulder pain will be evaluated through the NPRS scale. The participants will be asked to report their pain level on the NPRS (0–10 scale with 0 indicating no pain and 10 indicating the worst pain). The NPRS is a widely validated single 11-point numerical scale. The data obtained through NPRS is easily documented, intuitively interpretable, and meets regulatory requirements for pain assessment and documentation [21, 22].

#### Shoulder function measurement

Shoulder function will be evaluated using the SPADI questionnaire (Spanish validated version), which measures severity of upper extremity pain and disability during daily life activities [23]. This is a self-report questionnaire with 13 items on it (5 items specific to shoulder pain and 8 items specific to shoulder disability). The SPADI score can range from 0% indicating no pain or disability, to 100% indicating severe pain and total disability [24]. SPADI has been used previously in patients with DM [25, 26].

#### Shoulder’s range of motion (ROM)

The range of motion will be assessed through a digital inclinometer (Acumar™, Lafayette Instrument Company, Lafayette, IN). The inclinometer is reliable to measure the range of motion of shoulder flexion, abduction, scapular plane elevation, as well as internal and external rotation (ICC:0-85-0.97) [27]. For shoulder flexion, the arm should be actively elevated in a sagittal plane with the palm down until participants reach their full ROM, with the inclinometer placed on the distal upper arm proximal to the elbow, and distal to the glenohumeral joint. For shoulder abduction, the arm should be actively elevated in coronal plane with the thumb pointed upwards toward the ceiling until participants’ full ROM, with the inclinometer positioned on the distal arm proximal to the elbow, and distal to the glenohumeral joint. For shoulder elevation in scapular plane, participants will be instructed to perform elevation in scapular plane with the thumb pointing upwards toward the ceiling to full end range. The inclinometer will be positioned on the superior portion of the humeral shaft proximal to the elbow. To measure shoulder internal and external rotation, the participants will be in supine position with shoulder and elbow at 90° of abduction and flexion, respectively. The inclinometer must be placed on the distal dorsal surface of the forearm and the shoulder will be rotated actively in an internal and external rotation [28]. Three trials will be assessed for each movement.

#### Isometric shoulder muscles strength measurement

Isometric muscle strength of shoulder muscles will be assessed using a handheld dynamometer (HHD) (Lafayette Instrument, Lafayette, IN), a valid and reliable tool method (ICC = 0.94–0.97) [29–31]. Shoulder internal and external rotation strength will be evaluated following the protocol proposed by Kolber et al. [29], with the shoulder positioned in 0° of rotation, with the elbow positioned at a 90° of flexion and the wrist in neutral rotation. Shoulder flexion and abduction strength assessment will follow the protocol proposed by Çelik et al. [32] and Zanca et al. [33], respectively, with participants seated and the arm at 90° of elevation on a sagittal and scapular plane (30° anterior to the coronal plane). The dynamometer will be positioned in the distal humerus. Three repetitions of 5 s will be performed for each movement, with 30 s of rest between repetitions. A familiarization trial of each movement will be performed before data collection, to guarantee the participants’ comprehension [33]. The peak force (N) will be recorded for each trial and the mean of the three repetitions will be used for data analysis.

#### Shoulder three-dimensional kinematics measurement

Shoulder three-dimensional kinematics will be recorded during maximal arm elevation in the scapular plane and during the following functional tasks: hand to head (brush the hair), hanging clothes overhead, eating, and reaching an object overhead (at 120°). Each measurement will be repeated three times with 60-s intervals, following the protocol of Dogan et al. [34], using an inertial movement unit’s system (IMU system) consisting of a 3D accelerometer, a 3D gyroscope, and a 3D magnetometer (MVN AWINDA Starter Wireless, Xsens Technologies B.V. Enschede, Netherlands), which is already validated in the literature to measure 3D shoulder kinematics [35]. Eight sensors will be used and placed on the anterior sternum, on the back of the head, on the lateral aspect of the bilateral arms, on forearms and on wrists. Measurements were evaluated for individuals in an anatomical position, performing activity, and then returning to the anatomical position. Each measurement was repeated three times with 60-s intervals. The mean values of kinematic data determined each individual’s functional ROM of joints. The joint angle definitions followed by the International Society of Biomechanics [36]. Data processing will be performed through the MVN Analyze software (Xsens Technologies B.V. Enschede, Netherlands), and the MATLAB® software will be used for data processing (R2015b, Mathworks Inc., Natick, MA USA), considering all of the shoulder’s functional range of motion movements (flexion/extension; internal/external rotation; abduction/adduction).

#### Supraspinatus tendon thickness measurement

Ultrasonographic measurement for each participant’s supraspinatus tendon thickness will be scanned by using a Lumify Ultrasound in conjunction with a 4–12-MHz linear transducer (Philips Medical System) on musculoskeletal program. The protocol of the ultrasound measurement will be designed by an experienced sonographer in musculoskeletal ultrasound scanning. All ultrasound scanning will be performed by the researcher responsible of the study. Ultrasound images will be captured with the patients seated in an upright position, feet flat on the floor, neutral trunk posture, and head facing forward, evaluating supraspinatus tendon thickness in transverse and longitudinal, using both crass position and modified crass position [37–40]. The averaged value from the 3 measurements will be recorded as the supraspinatus tendon thickness.

#### AGE accumulation measurement

Skin autofluorescence is considered as a substitute variable of AGE accumulation. To perform this measurement, the AGE Reader (DiagnOptics, Groningen, the Netherlands) will be used, which non-invasively assesses skin autofluorescence. For this evaluation, participants will lay their arm on a special arm rest with a window in the middle of the AGE reader. A skin surface of around 4 cm^2^, guarded against surrounded light is illuminated with an excitation light source with LEDs of various intensities in a frequency range of 300 to 420 nm. Multiple photo diodes capture the auto fluorescent light, processes it, and generates the SAF (skin auto fluorescence) in arbitrary units (AUs). The resultant SAF is calculated as the ratio of the light intensity in a 420–600 nm wavelength range, and the light intensity in a 300–420 nm wavelength range. The assessment will follow the Meerwaldt et al. [41, 42] protocol. SAF measurements have been validated against biochemical analyses of skin biopsies in healthy participants, and in participants with type 1 or type 2 DM [25]. In participants with DM, SAF has an intra-individual, intra-day variability of 4.2–5.0%, and a seasonal variability of 5.9% [41, 43]. According to De Ranitz-Greven et al. [44], no increases were found in SAF postprandial, therefore a fasting measurement is not mandatory.

#### HbA1c (hemoglobin a1c), fasting glucose and lipid profile

HbA1c, fasting glucose, and lipid profile will be evaluated through blood tests that will be performed in a clinical laboratory, at the beginning (before starting the treatment protocol), after the 12-week intervention (3–7 days after the last session).

#### Global rating of change score

To evaluate the post-treatment results, focusing on the changes perceived by the participants.

### Plans to promote participant retention and complete follow-up {18b}

Researchers will be in contact with the participants during the study, through a telephone messenger group app, to solve doubts and share information. The participants will also be contacted by phone, twice/month to ask about their health status and to reinforce their participation.

### Data management {19}

Participants will receive a code number at the beginning of the study, which will be added in an excel file with all their personal information and measurements collected and analyzed (MFQ or RC), double checked (GZ, AC, or BS), and stored by the leading researcher (FHZ), protected with a password. Generated consents will be stored for 5 years after the end of the study, which will be discarded thereafter.

### Confidentiality {27}

The data collected can only be accessed by the responsible researcher and the other researchers (co-authors). Participants will receive a code number at the beginning of the study, only known by the responsible researcher, and will always maintain their anonymity in their study environment. All the data will only be used for academic purposes.

### Plans for collection, laboratory evaluation, and storage of biological specimens for genetic or molecular analysis in this trial/future use {33}

Not applicable, this study does not have biological specimens.

## Statistical methods

### Statistical methods for primary and secondary outcomes {20a}

Data will be analyzed using SPSS, version 20.0 (SPSS Inc. 233 S. Wacker Drive, Illinois USA). The statistical assumptions for all the tests will be assessed. In case of normality, to verify the effectiveness of protocols over time (12 weeks and follow-up of at week 20), a mixed model analysis of variance (ANOVA) with Bonferroni adjustment will be performed for pairwise comparisons. Variables that do not meet the ANOVA assumptions will be analyzed by the Mann-Whitney and Wilcoxon tests with Bonferroni correction a priori.

To assess the secondary objective of the study, Pearson’s or Spearman coefficient correlation, when applicable, and a linear regression analysis will be performed to assess the association between AGE accumulation and outcome measures.

The significance level will be set at 5% for all statistical analyses. Furthermore, the effect size will be calculated using the Cohen coefficient *d*.

### Interim analyses {21b}

Not applicable. Interim analyses will not be performed in the present study.

### Methods for additional analyses (e.g., subgroup analyses) {20b}

Not applicable. Additional analyses are not planned in the present study.

### Methods in analysis to handle protocol non-adherence and any statistical methods to handle missing data {20c}

The study will include only participants who attended at least 80% of the sessions and performed all the assessments (per-protocol). Furthermore, missing data will be handled by intention-to-treat analysis using the multiple imputation method.

### Plans to give access to the full protocol, participant-level data and statistical code {31c}

The datasets analyzed in the study may be requested from the corresponding author, considering if this agrees with the local research ethic committee.

## Oversight and monitoring

### Composition of the coordinating center and trial steering committee {5d}

The implementation of the trial is monitored by the researchers, composed by the main investigator (FAPHZ), which is the main liable entity for this trial and is also coordinator for the Laboratory of Clinical Research in Physical Therapy, and two other researchers responsible for the study organization, recruitment, and taking consent. In addition, there will always be two expert physical therapists in all interventions and assessment sessions. The researchers meet weekly for the appropriate course of study and to review trial conduct.

### Composition of the data monitoring committee, its role and reporting structure {21a}

A DCC committee is not necessary in this study, since the intervention protocol is of low risk and with a short duration. The research team is in charge of reporting immediately to the leading researcher (FHZ) about any inconvenience.

### Adverse event reporting and harms {22}

The procedures performed in the current study are not invasive. During and after clinical evaluations and treatment, the risks are minimal. A slight muscle discomfort may occur after evaluations and treatment sessions. For control, the patients will have their blood pressure, heart rate, and capillary glucose evaluated in all sessions. In the event of any adversity, the responsible researcher will provide the required assistance. Symptoms associated with the effects of the evaluated movements may also occur, such as fatigue and muscle pain. These are considered normal after physical exercise in the lower and upper limbs and which tend to last approximately 48 h. If the patient presents signs or symptoms such as glycaemia> 200 mg / dl, or, <80 mg / dl; systolic pressure> 180 mmHg, or diastolic pressure> 100 mmHg at rest, the session/evaluation will be immediately suspended, and they will be provided with the necessary assistance. All complications and dropouts will be reported in the final manuscript.

### Frequency and plans for auditing trial conduct {23}

No program to audit the trial will be performed, since this is a not invasive study, and it is of a short duration and has low risk. However, the Institutional Ethics Committee will review the trial conduct and final findings.

### Plans for communicating important protocol amendments to relevant parties (e.g., trial participants, ethical committees) {25}

In the case of any important protocol modification, the leading researcher will be responsible for reporting and submitting the new protocol version to the ethics committee for approval, as well as be in charge of updating the clinical registration trial.

### Dissemination plans {31a}

Considering the dissemination plans, in addition to disseminating the results with the patients themselves, the study is also expected to generate instances of constant linkage with the community through talks in care centers for diabetic patients, specialized hospitals in this field, and for physical therapists, when presenting the results of the study.

## Discussion

It is well stablished that hyperglycemia can negatively affect various systems, leading to cardiovascular disease, neuropathy, kidney disease, and other complications [3]. Regarding the impact of hyperglycemia on the musculoskeletal system, the literature shows that the higher HbA1c, higher the accumulation of AGEs, and the damage of connective tissues [4, 7]. Although the consequences of DM on the musculoskeletal system have been proved, patients, as well as the rehabilitation health service providers, in most cases, are not aware of the evidence linking DM to an increased risk of numerous musculoskeletal complications [45, 46]. Physical therapists should know that their patients with DM, especially those living with the disease for more than 10 years, or with poor glycemic control, are more likely to develop one or more musculoskeletal complications [45]. It must be considered that musculoskeletal disorders are usually associated with alterations in strength, mobility, and pain, which can result in a lower physical activity level and decreased social participation, adding important alterations in the individuals’ health and quality of life.

The impact of the DM on the musculoskeletal system is already well established [2–6]; however, most of the studies on shoulder musculoskeletal disorder rehabilitation focus on the general population, not considering specific protocols for DM individuals, or considering only one of the complications generated by DM, such as adhesive capsulitis [8, 9, 12, 47, 48]. Due to the abnormal collagen deposit in DM individuals, different musculoskeletal structures may be compromised, such as tendon and ligament injuries, making these connective tissues more susceptible to injuries [10, 43, 49].

Bearing in mind that DM is a highly prevalent disease and a worldwide public health problem, as well as the fact that upper extremity musculoskeletal disorders in DM are barely recognized and grossly underestimated, there is a clear need to investigate the effectiveness of treatment protocols that cover an improvement in these disorders. Most of the studies found in the literature used aerobic exercises, focusing exclusively on the cardiovascular system. It would be interesting to analyze if the combination of aerobic exercises and conventional musculoskeletal rehabilitation protocols could generate better results in functionality, pain, and mobility, as well as generate an improvement in the biochemical aspects related to the hyperglycemia of these patients, compared to the solely conventional musculoskeletal rehabilitation.

Therefore, the study presents an innovative purpose, since, in the literature, there are no randomized controlled trials evaluating the effectiveness of musculoskeletal treatment protocols for DM population, considering factors that are not only musculoskeletal, but also biomechanical and biochemical (AGEs). Provided protocols with proven effectiveness which are based on all the factors mentioned above could directly benefit not only patients with type 2 DM but also public health in general.

## Trial status

Version 1. November 1, 2021

Date recruitment begins: May 30, 2022.

Approximate date when recruitment will be completed: December 20, 2023.

## Data Availability

After study publication, the data and materials will be available upon a reasonable request to the corresponding author.

## Abbreviations

DM: Diabetes mellitus
AGEs: Non-enzymatic advanced glycation end-products
NPRS: Numerical pain rating scale
SRG: Shoulder-specific rehabilitation protocol group
SAG: Shoulder-specific rehabilitation protocol plus aerobic exercise group
HRR: Heart rate capacity
SPADI: Shoulder Pain and Disability Index
HbA1c: Hemoglobin a1c
ROM: Range of motion
HHD: Handheld dynamometer
N: Newton
IMU: Inertial Movement Unit
AU: Arbitrary unit
SAF: Skin auto fluorescence
